# Clinician-Prioritized Measures to Use in a Remote Concussion Assessment: Delphi Study

**DOI:** 10.2196/47246

**Published:** 2024-09-02

**Authors:** Keely Barnes, Heidi Sveistrup, Mark Bayley, Mary Egan, Martin Bilodeau, Michel Rathbone, Monica Taljaard, Shawn Marshall

**Affiliations:** 1 School of Rehabilitation Sciences Faculty of Health Sciences University of Ottawa Ottawa, ON Canada; 2 Bruyère Research Institute Ottawa, ON Canada; 3 Clinical Epidemiology Program Ottawa Hospital Research Institute Ottawa, ON Canada; 4 University of Ottawa, Faculty of Health Sciences, School of Human Kinetics Ottawa, ON Canada; 5 Carleton University, Systems and Computer Engineering Technology Ottawa, ON Canada; 6 Kite Research Institute Toronto Rehabilitation Institute University Health Network Toronto, ON Canada; 7 Division of Physical Medicine and Rehabilitation Temerty Faculty of Medicine University of Toronto Toronto, ON Canada; 8 Division of Neurology Department of Medicine, Faculty of Health Sciences McMaster University Hamilton, ON Canada; 9 School of Epidemiology and Public Health University of Ottawa Ottawa, ON Canada; 10 Department of Medicine University of Ottawa Ottawa, ON Canada

**Keywords:** telehealth, remote care, concussion, mTBI, mild traumatic brain injury, assessment, examination, telemedicine, remote care, TBI, traumatic brain injury, brain injury, Delphi, measure, measures, measurement, mobile phone

## Abstract

**Background:**

There is little guidance available, and no uniform assessment battery is used in either in-person or remote evaluations of people who are experiencing persistent physical symptoms post concussion. Selecting the most appropriate measures for both in-person and remote physical assessments is challenging because of the lack of expert consensus and guidance.

**Objective:**

This study used expert consensus processes to identify clinical measures currently used to assess 5 physical domains affected by concussion (neurological examination, cervical spine, vestibular, oculomotor, or effort) and determine the feasibility of applying the identified measures virtually.

**Methods:**

The Delphi approach was used. In the first round, experienced clinicians were surveyed regarding using measures in concussion assessment. In the second round, clinicians reviewed information regarding the psychometric properties of all measures identified in the first round by at least 15% (9/58) of participants. In the second round, experts rank-ordered the measures from most relevant to least relevant based on their clinical experience and documented psychometric properties. A working group of 4 expert clinicians then determined the feasibility of virtually administering the final set of measures.

**Results:**

In total, 59 clinicians completed survey round 1 listing all measures they used to assess the physical domains affected by a concussion. The frequency counts of the 146 different measures identified were determined. Further, 33 clinicians completed the second-round survey and rank-ordered 22 measures that met the 15% cutoff criterion retained from round 1. Measures ranked first were coordination, range of motion, vestibular ocular motor screening, and smooth pursuits. These measures were feasible to administer virtually by the working group members; however, modifications for remote administration were recommended, such as adjusting the measurement method.

**Conclusions:**

Clinicians ranked assessment of coordination (finger-to-nose test and rapid alternating movement test), cervical spine range of motion, vestibular ocular motor screening, and smooth pursuits as the most relevant measures under their respective domains. Based on expert opinion, these clinical measures are considered feasible to administer for concussion physical examinations in the remote context, with modifications; however, the psychometric properties have yet to be explored.

**International Registered Report Identifier (IRRID):**

RR2-10.2196/40446

## Introduction

Concussions also known as mild traumatic brain injuries can occur in a variety of different settings such as within sports, in the workplace, and from falls that occur during activities of daily living [1]. A diverse group of clinicians including sports medicine physicians, neurologists, physiatrists, and physiotherapists may complete physical assessments post concussion. Approaches to completing the concussion physical examination appear to be variable among clinicians. While the available guidelines provide an overview of the important components of a vocational assessment following a concussion [2], there are no widely agreed-upon guidelines outlining specific measures to use in the concussion assessment. This is particularly important in the context of work-related injuries where benefits and treatment are related to clinical findings. Roughly 1 in 5 adults are unable to return to work at 6 months post injury following their concussion [1], many of whom require specialized assessment at this time point post injury.

Several domains, each with various potential measures, should be assessed in the concussion examination [2]. Furthermore, different measures may be used when the person who is injured is evaluated immediately following a concussion injury, a few days following the injury, and a few months following the injury. Leddy et al [3] outlined an evidence-based physical examination for neurologists assessing adults presenting with persistent symptoms post concussion; however, specific measures for certain domains remain undefined.

Identification of appropriate measures for remote concussion assessment is an important challenge that moved to the forefront of health care with the COVID-19–driven shift in clinical practice to remote care. Remote concussion assessments are increasingly common; yet, the measures used to complete these assessments continue to vary due to the lack of standardization and consistency regarding best practices among clinicians. In response to this shift, remote examination resources targeting family medicine physicians have been developed to support the diagnosis and acute evaluation of concussion through remote assessment [4]. These resources outline approaches that can be transferred from the typical in-person concussion examination to a remote context. Specific limitations include orthostatic vitals, dynamic gait, and cardiovascular or respiratory evaluation which are very difficult to complete in a remote context without special equipment being sent to the patient. Also, in response to the shift to remote care, McPherson et al [5] published adaptations to the evaluation of cranial nerves, oculomotor, vestibular, and cervical spine deficits along with an assessment of orthostatic intolerance from the Buffalo Concussion Physical Examination for use in the remote assessment of sport outpatients (in both acute and persistent symptom contexts). Finally, a living guideline for the acute assessment and management of pediatric concussion presents approaches to completing the remote concussion examination in the pediatric population [6]. These resources are based on expert opinion and support the feasibility of completing components of the concussion examination remotely. Variations in the approaches to remote assessment presented in the above-noted resources highlight the lack of standardized approaches to the remote assessments currently used in clinical practice. Furthermore, most of these resources were developed in the context of the sports setting or the pediatric patient population. Information is lacking on the measures recommended for use in remote concussion examination of community-dwelling adults who may have sustained a concussion injury in the workplace and are experiencing persistent symptoms.

In the absence of evidence or sufficient information to support clinical decisions, such as decisions on clinical measures to use in practice, expert consensus is often sought [7,8]. Further, 1 approach to finding consensus on possible clinical measures is to use the Delphi method which typically involves at least 2 rounds of survey administration to expert clinicians. Commonly, exploratory questions are asked in the first round followed by more targeted, specific questions in a subsequent round. The Delphi method is a useful approach for obtaining anonymous opinions from several participants across disciplines and wide-spread locations [7]. Expert opinions on the topic of interest transform into consensus in this approach [9]. Importantly, consensus does not indicate that the measures selected are the correct measures to use in a concussion assessment. Rather, the results from the consensus act to structure follow-up discussions (in working groups) and inform subsequent decisions [10].

Previous work has used the Delphi approach to identify clinical measures in various populations. Reneker et al [11] aimed to document the cervical spine measures used in concussion to distinguish between cervicogenic and other factors contributing to dizziness postsport-related concussion. Consensus on the clinical utility of the identified measures was determined by the clinicians who participated in this study. Similarly, while not specific to concussion, Winser et al [12] used a Delphi approach to identify balance measures used by clinicians to evaluate people with cerebellar ataxia. Clinicians were first asked to list the measures that they use in practice and then select the most appropriate measure for the assessment of balance. This study identified 3 balance measures (Berg Balance Scale, the scale for the assessment and rating of ataxia, and the Timed Up and Go) as most appropriate; however, it noted that an evaluation of test psychometric properties is needed. While these studies identify balance and cervical spine measures for specific purposes, there remains limited information on the identification of clinical measures to use in the evaluation of physical domains impacted by a concussion.

The purpose of this work was to identify clinical measures currently used to assess 5 predefined domains following a concussion injury (neurological examination, vestibular, oculomotor, cervical spine, and effort); and determine the feasibility of administering the measures remotely. Findings from this study will inform the selection of measures for use in a remote concussion assessment that will be tested in a planned evaluative study. We focused on the physical domains of a concussion examination since they appear to be more challenging and require more adaptations to administer virtually when compared to measures of cognitive or emotional health. Further, while there is overlap in concussion symptom domains, classification systems typically segregate physical symptoms from cognitive and emotional symptoms. For example, Ellis et al [13] presented a classification system in which signs and symptoms post concussion are grouped into physiologic, vestibulo-ocular, cervicogenic, and cognitive or mood-related domains.

## Methods

### Study Design

The complete methodology is presented in a study by Barnes et al [14]. The published protocol presents a flow diagram of the methodological process, which includes a round 1 survey to identify clinical measures used to evaluate physical domains impacted by a concussion, a round 2 survey to rank-order the identified measures from round 1, and a working group to document the perceived feasibility of administering the identified measures from the surveys in a virtual environment.

### Delphi Survey Approach

#### Round 1

An open invitation was sent to clinician-members of 8 regional and 4 national brain injury or concussion professional associations or networks through email and monthly newsletters over 3 months (February to May 2022). Wherever possible, targeted emails to clinicians with publicly available contact information were used to ensure there was a representative sample of at least 2 participants from each clinical profession of interest (physiatry, sports medicine, neurology, and physiotherapy). Purposive sampling techniques were used to recruit at least 50 expert Canadian-practicing clinicians with experience completing in-person concussion assessments in any setting (sports contexts, emergency departments, rehabilitation centers, family medicine offices, etc). Justification of the sample size is presented in the published protocol [14]. As reported by Hasson et al [10], the inclusion of participants who have an interest in the topic and knowledge about the topic may aid in increasing the content validity of the findings of the Delphi approach. Through the open invitation and self-reports of competency, we were able to recruit participants who were interested in concussion assessment and management and who had sufficient experience completing concussion assessments, potentially strengthening the content validity of the survey findings.

A comprehensive list of clinical measures used to assess 5 domains of concussion (neurological examination, vestibular, oculomotor, cervical spine, and effort assessment) at all time points post injury (acute, subacute, or persistent) was compiled. An “other” domain was also included in round 1 of the survey due to the anticipated classification variations between clinicians for certain clinical measures. For example, balance or gait tests were included in the vestibular as well as in the “other” domain by clinicians. An adjusted list of measures was created to group all clinical measures under the predetermined domains. The adjusted list of measures was rank ordered by frequency. Only measures that reached at least 15% agreement ([number of participants that identified measure total number of participants] 100) were included in round 2. Due to the number of measures anticipated to be identified in the survey, we previously set the 15% criteria so that we could narrow down the most relevant measures in the subsequent round, with a relatively good level of agreement among clinicians. Further, to ensure representation from each clinical profession, we planned to include any measures reaching at least 60% agreement with a professional group in round 2. All participants who completed round 1 were invited to complete round 2. To ensure the survey responses were anonymous, in line with the Delphi approach, a separate form was created for email input so that we could send the round 1 participants the round 2 survey.

#### Development of Reading Materials (Review of the Literature)

As we were particularly interested in the accuracy with which the tools identified pathology, a literature review of the sensitivity and specificity of retained measures was carried out following round 1 using the following databases: PubMed (Ovid; National Library of Medicine), MEDLINE (Ovid), CINAHL (Cumulative Index to Nursing and Allied Health Literature; EBSCO Information Services), and Google Scholar. Search terms used were related to the population of interest (all brain injuries due to limited information in the literature on psychometric properties in concussion), psychometric properties of interest (sensitivity and specificity), and clinical measures of interest (measures presented in Table 1). No date limitations were set. The findings from the review of the literature were used to develop descriptions of the identified measures and their psychometric properties; this information was provided in written form to all round 2 participants. The measures’ sensitivity and specificity metrics identified from the review of the literature that were used to develop the reading materials provided to round 2 participants are presented in Multimedia Appendix 1 [12,15-27].

**Table 1:** Round 1: list of measures that reached at least 15% agreement (N=58).

Domain and measures		Frequency, n (%)
**Neurological examination**		
	Coordination: finger-to-nose or heel-to-shin; rapid alternating movements	37 (64)
	Cranial nerve	34 (59)
	Sensation	20 (34)
	Reflexes	19 (33)
	Motor (tone, pronator drift, or strength or power using MRC^a^ grading or MMT^b^)	16 (28)
	Myotomes	13 (22)
**Vestibular**		
	VOMS^c^	25 (43)
	Balance (feet together, single leg stance, or tandem stance)	23 (39)
	VOR^d^ assessment	21 (36)
	BESS^e^ or mBESS^f^	21 (36)
	Dix-Hallpike	20 (34)
	Head thrust or head impulse test	14 (24)
	Gait or tandem gait	13 (22)
	Romberg	12 (20)
	Dynamic visual acuity	10 (17)
**Oculomotor**		
	Saccades	21 (36)
	Convergence	20 (34)
	Smooth pursuits	15 (26)
**Cervical**		
	Range of motion	49 (84)
	Palpation	26 (44)
	Strength (MMT or DNF^g^ endurance)	21 (36)
	Joint position error test	9 (16)

^a^MRC: medical research council.

^b^MMT: manual muscle testing.

^c^VOMS: vestibular ocular motor screening.

^d^VOR: vestibulo-ocular reflex.

^e^BESS: balance error scoring system.

^f^mBESS: modified balance error scoring system.

^g^DNF: deep neck flexor.

#### Round 2

The expert clinicians were provided with the results of round 1 and reading materials before completing the second-round surveys and were given a month to complete the second survey. Round 1 results were summarized in tables outlining all measures that were at or above the 15% level of agreement and all additional measures that did not meet the cutoff. The sensitivity, specificity, and additional considerations for the administration of each measure (eg, equipment needed or time for administration) identified in the review of the literature were presented. The clinicians were then asked to rerank the identified measures from most to least relevant to their in-person clinical practice. Mean rankings were calculated by summing the product of the weight and frequency count for each measure and dividing by the total number of responses. The weight was determined by the number of measures within each domain.

#### Working Group

A working group, consisting of a physiatrist, neurologist, physiotherapist-researcher, and sports medicine physician, met in June 2022 to determine the feasibility of administering the measures virtually. The working group members were recruited through targeted emails. A moderator (KB) facilitated the working group. The group was audio and video recorded, transcribed into a written format, and analyzed using content analysis. A physiatrist (M Bayley; member of the working group but did not attend working group discussion) reviewed the measures independently and commented on the feasibility of the remote application of the measures based on personal experience and expertise.

### Ethical Considerations

This study was approved by the Ottawa Health Sciences Network Research Ethics Board (20210575-01H), the Bruyère Research Institute Research Ethics Board (M16-22-006), and the University of Ottawa Board of Ethics (H-02-22-7611).

## Results

### Round 1

**Figure 1:**
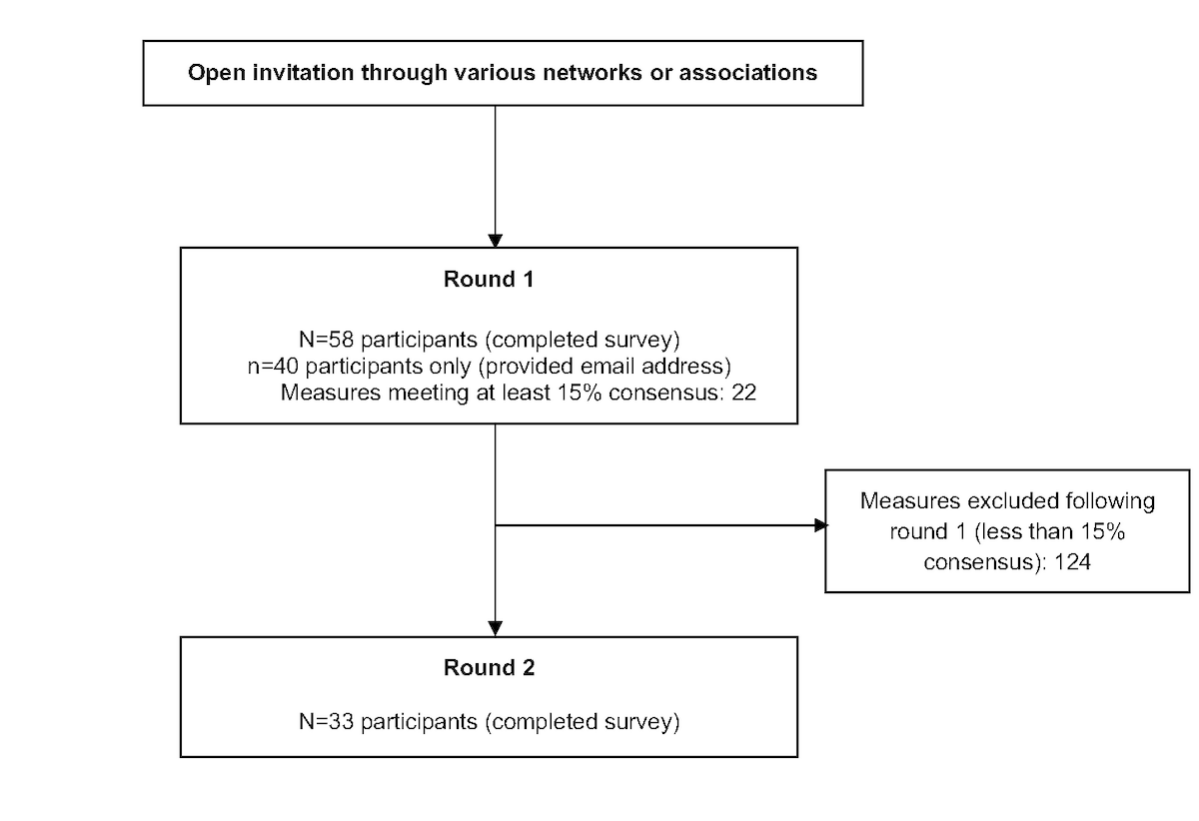
Delphi survey flow diagram.

Figure 1 presents a flow diagram of the survey process. In total, 59 clinicians participated in round 1 of the Delphi survey. The demographic information of the included participants is presented in Table 2. Most participants were physiotherapists followed by sports medicine physicians, physiatrists, and neurologists. The “other” category of clinical professions included 5 occupational therapists, 3 kinesiologists or athletic therapists, 2 speech-language pathologists, 2 trainees (medical and physiotherapy), a physician’s assistant, an orthopedic surgeon, and a family medicine physician. The majority of participants had over 10 years of experience in clinical practice and assessed over 50 people with concussions annually. Further, 1 participant self-reported as “strongly incompetent” for their competency of completing the in-person concussion assessment, and, therefore, their responses were excluded from data analysis as the aim was to survey experts leaving 58 participants in round 1.

**Table 2:** Demographic information of clinicians that completed round 1 survey (N=58).

		Frequency, n (%)
**Age (years)**		
	20-29	10 (17.2)
	30-39	25 (43.1)
	40-49	8 (13.8)
	50-59	10 (17.2)
	60+	5 (8.6)
	Prefer not to respond	0 (0)
**Clinical profession**		
	Physiatrist	5 (8.6)
	Neurologist	2 (3.4)
	Sports medicine physician	10 (17.2)
	Physiotherapist	26 (44.8)
	Other	15 (25.9)
**Clinical practice (years)**		
	1-5	17 (29.3)
	5-10	13 (22.4)
	10+	27 (46.6)
	Did not respond	1 (1.7)
**Volume of practice (patients with concussion assessed per year)**		
	0-25	20 (34.5)
	25-50	16 (27.6)
	50+	22 (37.9)
**Self-report competency in-person**		
	Strongly incompetent	0 (0)
	Incompetent	0 (0)
	Neutral	10 (17.2)
	Competent	31 (53.4)
	Strongly competent	17 (29.3)
**Self-report competency virtual**		
	Strongly incompetent	2 (3.4)
	Incompetent	6 (10.3)
	Neutral	24 (41.4)
	Competent	23 (39.7)
	Strongly competent	3 (5.2)

After categorizing the measures identified in round 1 based on the definitions of the domains, 146 different measures were identified (31 measures were identified in the neurological examination domain, 41 were identified in the vestibular domain, 26 in the oculomotor domain, 37 in the cervical spine domain, and 11 in the effort domain). The measures that reached the predefined cutoff level of agreement (at least 15%) are presented in Table 1. The list of clinical measures that did not meet the 15% cutoff is presented in Multimedia Appendix 2. The majority of clinicians reported not using any measures to evaluate effort, malingering, or symptom validity in their clinical practice. No relevant measures met the cutoff criteria for the effort domain, and it was, therefore, not included in the second survey. Further, when responses were grouped and analyzed based on the clinical profession (physiotherapist, neurologist, physiatrist, or sports medicine physician) of the respondents, no additional measures met the criteria of at least 60%.

### Round 2

In total, 33 of the 40 clinicians who agreed to be contacted for round 2 completed this survey. Table 3 presents the mean rankings for each of the measures and presents the final rank orders for each of the domains. Multimedia Appendix 3 includes all ranking values, weights, and frequency counts. Multimedia Appendix 4 includes consensus values for round 2.

**Table 3:** Round 2: mean rankings of measures.

Domain and measures			Mean ranking
**Neurological examination (mean ranking /6)**			
	Coordination: finger-to-nose or heel-to-shin; rapid alternating movements	4.67	
	Cranial nerve (evaluation of 12 cranial nerves)	4.52	
	Motor (tone, pronator drift, or strength or power using MRC^a^ grading or MMT^b^)	4.09	
	Reflexes	2.85	
	Myotomes	2.45	
	Sensation	2.42	
**Vestibular (mean ranking /9)**			
	VOMS^c^	7.52	
	Balance (feet together, single leg stance, or tandem stance)	6.45	
	VOR^d^ test	6.18	
	BESS^e^ or mBESS^f^	5.61	
	Dix-Hallpike	4.58	
	Gait or tandem gait	4.36	
	Head thrust or head impulse test	3.91	
	Dynamic visual acuity	3.52	
	Romberg	2.88	
**Oculomotor (mean ranking /3)**			
	Smooth pursuits	2.24	
	Saccades	1.91	
	Convergence	1.85	
**Cervical (mean ranking /4)**			
	Range of motion	3.67	
	Palpation	2.94	
	Strength (MMT or DNF^g^ endurance)	1.88	
	Joint position error test	1.52	

^a^MRC: medical research council.

^b^MMT: manual muscle testing.

^c^VOMS: vestibular ocular motor screening.

^d^VOR: vestibulo-ocular reflex.

^e^BESS: balance error scoring system.

^f^mBESS: modified balance error scoring system.

^g^DNF: deep neck flexor.

### Working Group

All participants in the working group had over 10 years of experience in clinical practice and self-reported as “competent” or “strongly competent” in completing in-person concussion assessments. Only 1 working group participant self-reported a neutral competency in completing the remote assessment; however, all participants had sufficient experience with conducting remote concussion assessments. All others self-reporting as “competent” or “strongly competent.” Reports of competency were subjective and based on self-perception.

When asked what technologies were used to complete remote assessments, participants reported using either a laptop, desktop, or laptop with a large external monitor. Further, 3 participants reported not always being able to see the whole body of the patients and only 1 participant reported being able to see the whole body of patients when completing assessments when requested. The physiatrist who reviewed the measures independently identified similar concerns to those of the members of the working group.

In total, 16 measures were deemed feasible to complete virtually but of these, the majority would require modifications (see Tables 4-7). Further, 6 measures were deemed infeasible to complete virtually.

**Table 4:** Working group: feasibility of completing identified neurological examination measures virtually as reported by the working group members.

Domain and measures		Considerations of administering a measure virtually as identified by experts	Feasible when administered virtually?
**Neurological examination**			
	Coordination: finger-to-nose or heel-to-shin; rapid alternating movements	Need to see end point (stable end point) for finger-to-nose: touch camera on screen Heel-to-shin is much more challenging due to the positioning of the camera: possible if someone is holding the camera Rapid alternating movements is feasible	Yes^a^
	Cranial nerve	Taste and smell are a challenge (would see the object before smelling) Use of a Snellen chart is a challenge Consistency of instructions for administration is a barrier	Yes (for the majority)
	Motor (tone, pronator drift, or strength or power using MRC^b^ grading or MMT^c^)	Tone is not feasible Pronator drift is feasible Manual muscle testing is feasible up to 3/5 Cannot complete traditional power testing of muscles Feasible if incorporating a functional test (squat) to provide insight into strength	Yes (for the majority)
	Reflexes	Only feasible if a trained clinician is with the person in-person	No^d^
	Myotomes	Feasible up to 3/5 grading	Yes, but not recommended
	Sensation	Could self-assess but may not be useful information Risk of false negative is high: not recommended virtually	Yes with modifications, but not recommended

^a^Yes: measure is feasible when administered virtually.

^b^MRC: medical research council.

^c^MMT: manual muscle testing.

^d^No: measure is not feasible when administered virtually.

**Table 5:** Working group: feasibility of completing identified vestibular measures virtually as reported by the working group members.

Domain and measures		Considerations of administering a measure virtually as identified by experts	Feasible when administered virtually?
**Vestibular**			
	VOMS^a^	Smooth pursuits, saccades, VOR^b^: feasible (most clinicians only complete these 3 components); need a well-placed camera VOR: feasible, but keeping at the right speed is a challenge; could use a metronome smartphone app Convergence: feasible, measurement is the barrier (unless having a person helping in-person) Visual motion sensitivity: feasible; however, safety concern if standing, need to have enough range (cannot do this in seated position unless while having a swivel chair) If solely using VOMS to capture subjective changes in symptoms, then it would be feasible	Yes^c^ with modifications
	Balance (feet together, single leg stance, or tandem stance)	The challenge is obtaining a full view of the person completing the test Concerns regarding safety, especially with single-leg stance Modify environment: have a table in front to grab on to, a couch nearby to fall into, another person in-person	Yes
	VOR test	The challenge is seeing eye movements Feasible if evaluating subjective response	Yes
	BESS^d^ or mBESS^e^	Full BESS would not be feasible due to safety concerns when standing on a foam surface mBESS may be feasible; however, safety is still a concern Seeing errors may be a challenge depending on the camera angle	Yes (mBESS only)
	Dix-Hallpike	Not feasible unless someone trained is with the person in-person	No^f^
	Head thrust or head impulse test	Not feasible as the movement must be unexpected Neck pain is a barrier even in-person	No
	Gait or tandem gait	Feasible; however, safety is a concern Modify the environment (couch or wall nearby) or have another person in-person Camera angle is a challenge: prefer profile view when observing gait	Yes
	Dynamic visual acuity	The computerized dynamic visual acuity test may be feasible; however, there is a need for specialized equipment on the head, likely making it unfeasible Clinical dynamic visual acuity requires a specific set-up (Snellen chart or specific distance from chart), identifying the objective difference would be a challenge and therefore likely not feasible Requires a person in-person	No
	Romberg	Feasible; however, safety is a concern and may not be sensitive to balance deficits, especially if administered virtually	Yes

^a^VOMS: vestibula ocular motor screening.

^b^VOR: vestibulo-ocular reflex.

^c^Yes: measure is feasible when administered virtually.

^d^BESS: balance error scoring system.

^e^mBESS: modified balance error scoring system.

^f^No: measure is not feasible when administered virtually.

**Table 6:** Working group: feasibility of completing identified oculomotor measures virtually as reported by the working group members.

Domain and measures		Considerations of administering a measure virtually as identified by experts	Feasible when administered virtually?
**Oculomotor**			
	Smooth pursuits	Feasible, person follows finger on the screen, could use YouTube videos with object moving to standardize	Yes^a^
	Convergence	Feasible; however, measurement is a challenge	Yes with modifications
	Saccades	Feasible, look at the top corner of 1 side of the screen to the top corner of the other side	Yes

^a^Yes: measure is feasible when administered virtually.

**Table 7:** Working group: feasibility of completing identified cervical spine measures virtually as reported by the working group members.

Domain and measures			Considerations of administering a measure virtually as identified by experts		Feasible when administered virtually?
**Cervical**					
	Range of motion	Only feasible for active range of motion Feasible, side view for neck flexion and extension, anterior view for lateral flexion, rotation is a challenge (could use a smartphone app to detect angle for rotation) The challenge is not being able to complete passive evaluation		Yes^a^ with modifications	
	Palpation	Feasible if self-palpate Subjective but useful information		Yes with modifications	
	Strength (MMT^b^ or DNF^c^ endurance)	DNF endurance: not feasible (camera angle would be a challenge, person needs a specific positioning of the neck which would be a challenge to obtain virtually, usually need a hard surface) MMT: can obtain an idea of up to 3/5 grading, could complete self-resistance May not be useful for concussion		No^d^	
	Joint position error test	Not feasible, specialized equipment and specific positioning are needed Could use a modified version where the person looks at the light on the computer and the clinician monitors for repositioning of the head May not be generalizable or useful as a screening assessment		No	

^a^Yes: measure is feasible when administered virtually.

^b^MMT: manual muscle testing.

^c^DNF: deep neck flexor.

^d^No: measure is not feasible when administered virtually.

A discussion regarding the sensitivity and specificity of the balance measures occurred during the working group deliberations. Specifically, some clinicians reported that based on their experience with in-person administration, remote administration of certain measures, such as coordination assessments, provided less reliable information. The balance error scoring system or modified balance error scoring system was judged by them to have a poor ability to distinguish between healthy participants and participants with concussion. The working group participants; however, reported that the modified balance error scoring system and Romberg may be useful tools to screen for gross motor balance deficits in a remote examination. These measures were, therefore, deemed feasible and potentially useful to include in the remote concussion assessment for people presenting with persistent symptoms. Based on the round 2 survey results, balance testing (single leg stance, tandem stance, or feet together) and vestibular ocular motor screening (VOMS) may be more relevant to clinical practice and therefore more suitable to include in the remote assessment toolkit. Similarly, the clinicians reported that the objective measurement of convergence in the convergence oculomotor measure and the near point convergence component of the VOMS are extremely challenging to assess in a remote context; however, it could be a useful tool if solely using the test as a screening tool to document subjective changes in symptoms. Some clinicians reported that the amount of reliable information that they receive with remote administration of the VOMS is minimal; however, components of the test may be useful.

## Discussion

### Principal Findings

In this study, we used a Delphi survey methodology to identify all clinical measures clinicians use to evaluate the physical domains impacted by a concussion injury at all time points post injury. This was followed by an expert-clinician working group that documented the current perceived feasibility and use of the measures for remote assessments.

This study confirmed that clinicians use varied approaches when completing in-person evaluations of the physical domains impacted by a concussion injury with almost 150 different concussion evaluation measures identified by the survey clinician-participants. Based on the findings, it appears that there is no widely accepted, standardized approach to assessing concussions; however, the use of coordination testing, the VOMS, smooth pursuits, and cervical spine range of motion may be the most relevant measures. This is therefore a prime area for some standardization work. Matuszak et al [28] documented that the concussion examination in still-symptomatic participants has not yet been standardized or supported by evidence, and the variation in measures identified in this study may reflect this lack of standardization. According to the Living Concussion Guidelines, valid, standardized tools are needed to assess and monitor symptoms [29]; however, specific measures containing these characteristics have not yet been outlined. Concussions are a unique condition in which assessments are conducted in a variety of different locations and by a heterogeneous group of clinicians [3]. This, therefore, may have contributed to the variety of measures identified by the clinicians who participated in the survey. Further, many factors may have contributed to the selection of measures by the clinician-participants such as time since injury, clinician practice type, cost of clinical measures, equipment needed, ease of use, time needed to administer a measure, accessibility, familiarity, among others [30,31].

It is possible that the reading material provided to the participants outlining the psychometric properties of the tools based on the review of the literature did not influence the responses and rankings in the second-round survey resulting in a maintenance in ranking of measures from round 1 to round 2. Presenting the reading material, which included information about the measure properties, may have reinforced the participants’ original opinions about the clinical measures, leading clinicians to rank the most common measures from round 1 as first in round 2. Reasoning for this may be due to the psychometric properties associated with the measures. As demonstrated in Multimedia Appendix 1 [12,15-27], the documented psychometric properties are commonly weak to moderate or undefined for many of the measures used in a concussion assessment whether performed virtually or in-person. Clinician rankings for coordination testing, VOMS and cervical range of motion in round 1 and round 2 (within their respective domains) did not change, where all 3 measures were found to have acceptable sensitivity properties (above 70%); however, the properties available for coordination testing and cervical range of motion are not specific to concussion. Stokes and O’Neill [32] documented that physiotherapists are aware of how to administer clinical measures and track progress using the measures; however, their confidence is lower in terms of their knowledge regarding the properties of the measures. Wedge et al [33] similarly reported that physiotherapists need more information on the psychometric properties of clinical measures. Most physiotherapists included in the study by Wedge et al [33] indicated that they did not critically assess the properties of the clinical measures they use in their practice; however, there is a desire to have this information more readily available. Many of the physiotherapists further reported that they were unaware of publications providing relevant psychometric property information [33]. Awareness and knowledge of the relevant psychometric properties may therefore inform the selection of clinical measures. In practice, clinicians are encouraged to adopt an approach to assessment that is evidence-based, which includes consideration of the current research, clinical expertise, and client or patient preference [34]. There are, however, challenges and barriers to implementing evidence-based practice in clinical settings, such as time limitations, and lack of access to up-to-date research [34]. These factors may have further influenced the clinicians’ selections of measures in the surveys in this study. Clinicians selecting measures without the information provided in this study may have come to different, less evidence-based ranking decisions.

The measures identified in the surveys and discussed in the working group are in line with the recommendations presented in concussion assessment and management guidelines and remote concussion resources [2-4,6]. Guidance for the remote general neurologic examination was presented by Al Hussona et al [35] and aligns with the findings of the working group in this study. According to this guidance, the majority of the cranial nerve examination can be feasibly completed virtually, including the motor (muscle bulk, pronator drift, antigravity power, or squat), sensory (compare light touch or cold on both index fingers and tops of both of the big toes), coordination (including finger-to-nose, heel-to-shin, and rapid alternating movements), and gait (that includes double leg stance, normal gait, and tandem gait) examinations. Similarly, according to Ellis et al [6], a modified cranial nerve examination (in the concussion context) can be completed virtually and includes evaluation of extraocular movements, smooth pursuits, facial symmetry, facial sensation, and movement of palate and tongue. Motor and coordination evaluation according to this guideline includes pronator drift assessment and rapid alternating hand movements. Some limitations identified by Al Hussona et al [35] for the remote examination include the inability to perform a fundoscopy examination, detailed power examination, and neuro-otology maneuvers such as the Dix-Hallpike. While the guidance by Al Hussona et al [35] is not specific to the concussion examination, it does support the findings of the working group in this study per the concussion assessment as the concerns and considerations that were brought up by the working group members are similar to those presented in the guidance.

Montes et al [36] developed a roadmap for remote assessment in neuromuscular disorders, and this roadmap could be used across various conditions, including concussion. According to Montes et al [36], effective adoption of measures in remote practice should include identifying measures that are clinically relevant and patient-centered. Further, expert opinion and consensus are needed to identify the feasibility of remote assessments in various populations and conditions. Measures that could be safely and feasibly administered in remote environments should be the first point of consideration for adaption to remote environments. Guided by this work by Montes et al [36], the findings from this study provide an informed foundation on which to base the remote concussion assessment. It is important to note that this study identifies the clinician-perceived measures that are most relevant to assessing certain physical domains affected by concussion [3]. By identifying the most relevant measures (and some of their associated psychometric properties) used to evaluate the physical domains impacted by a concussion injury that are feasible virtually, we now have an understanding of the clinician-perceived measures that should be initially explored when commencing to determine measures that should be administered in the different contexts, both in-person and virtually.

### Limitations

The majority of participants in the Delphi survey were physiotherapists and there was an uneven distribution of participants based on clinical profession. Physiotherapists’ opinions may, therefore, be relatively overrepresented in this survey compared to the other clinical professions. To address this, we included a cutoff of consensus among each clinical profession as well as among all participants; however, with only 2 neurologist, 2 speech-language pathologist, and 3 kinesiologist or athletic therapist responses, reaching that cutoff would be a challenge for those professions. Similarly, while the working group included representation from clinical professionals who typically complete a concussion examination, input may be missing from other clinical professions such as athletic therapists, trainers, or kinesiologists, nurses, and neurosurgeons. Furthermore, concussion injuries may occur in different contexts and may be assessed by different clinician professionals and at different time points; these factors may contribute to the low level of consensus and the broad range of measures identified in both rounds of surveys in our study. There may have been greater consensus if the questions were specific to assessments conducted on people experiencing persistent symptoms post injury; however, based on the lack of guidance available on the specific measures to use in the assessment of people experiencing persistent symptoms, variation would be expected.

### Conclusion

The Delphi approach resulted in low to moderate agreement among the clinician-participants regarding the most relevant measures used to evaluate certain physical domains impacted by a concussion injury. This work highlights the broad range of measures being used in practice; however, it points to the use of coordination testing, the VOMS, smooth pursuits, and cervical spine range of motion as measures that may be most relevant in a concussion examination, within their respective domains. These measures ranked first among the survey clinician-participants determined to be feasible with modifications in a remote context as decided upon by expert clinicians in the working group. While these measures have been included in most of the remote concussion assessment guidelines and resources, the psychometric properties are underdeveloped for all in the concussion context. These measures, therefore, present a clear starting point for the investigation of psychometric properties associated with remote administration given their rankings and perceived feasibility in the remote environment.

## Appendix

Multimedia Appendix 1 List of measures with identified psychometrics that reached at least 15% agreement in round 1.

Multimedia Appendix 2 Delphi survey round one: measures that did not meet the 15% cut-off criteria.

Multimedia Appendix 3 Round 2 mean rankings.

Multimedia Appendix 4 Round 2 consensus values.

## Abbreviations

CINAHL: Cumulative Index to Nursing and Allied Health Literature
VOMS: vestibular ocular motor screening

## Data Availability

All data generated or analyzed during this study are included in this published paper.
