# Supplementary material for: Clinician-Prioritized Measures to Use in a Remote Concussion Assessment: Delphi Study
Source: JMIR Form Res. 2024 Sep 2;8:e47246. doi: 10.2196/47246 (PMC11406108; doi:10.2196/47246)
Supplement: Multimedia Appendix 1 [file formative_v8i1e47246_app1.docx]

**Multimedia Appendix 1**

**Table S1.** List of measures with identified psychometrics that reached at least 15% agreement in round 1 (N=58).

| Domain | Measures | Diagnostic Accuracy |
| --- | --- | --- |
| Neurological Examination | Coordination: finger-to-nose, heel-to-shin; Rapid alternating movements | Adults with stroke:  Timed Finger to nose:  **Sensitivity:** 71%  **Specificity:** 69%  [1] |
|  | Cranial nerve | Adults with focal cerebral hemisphere lesions:  Visual field defect:  **Sensitivity**: 22%  **Specificity**: 95%  [2] |
|  | Sensation | Not reported |
|  | Reflexes | Adults with focal cerebral hemisphere lesions:  Hyperreflexia:  **Sensitivity**: 11%  **Specificity**: 100%  [2] |
|  | Motor (tone, pronator drift, strength/power using MRC grading/MMT) | Adults with focal cerebral hemisphere lesions:  Pronator drift:  **Sensitivity:** 22%  **Specificity:** 100%  Focal muscle weakness:  **Sensitivity**: 20-30%  **Specificity:** 100%  [2] |
|  | Myotomes | Not reported |
| Vestibular | VOMS | Adults with acute/subacute concussion:  **Sensitivity:** 96%  **Specificity:** 46%  [3] |
|  | Balance (feet together, single leg stance, tandem stance) | Community-dwelling older adults with recurrent falls:  Single leg stance:  **Sensitivity:** 33%  **Specificity:** 71%  [4] |
|  | VOR assessment | Not reported |
|  | BESS/mBESS | Adults with acute concussion:  **Sensitivity:** 10-60%  **Specificity:** 40-93%  [5, 6] |
|  | Dix-Hallpike | Adults with benign paroxysmal positional vertigo:  **Sensitivity:** 82%  **Specificity:** 71%  [7] |
|  | Head thrust/Head impulse test | Adults with vestibular hypofunction:  **Sensitivity:** 63-72%  **Specificity:** 64-78%  [8] |
|  | Gait/Tandem gait | Youth with acute/subacute concussion:  Tandem gait:  **Sensitivity:** 41%  **Specificity**: 90%  [9] |
|  | Romberg | Young adults with vestibular dysfunction:  **Sensitivity:** 55%  **Specificity:** 64%  [10] |
|  | Dynamic Visual Acuity | Not reported |
| Oculomotor | Saccades | Youth and adults with TBI:  Computerized only:  Horizontal:  **Sensitivity:** 77%  **Specificity:** 78%  Vertical:  **Sensitivity:** 64%  **Specificity:** 65%  [11] |
|  | Convergence | Not reported |
|  | Smooth pursuits | Youth and adults with TBI:  Computerized only:  **Sensitivity**: 68% **Specificity:** 73%  [12] |
| Cervical | Range of motion | Adults with whiplash:  Computerized:  **Sensitivity:** 86%,  **Specificity:** 95%  [13] |
|  | Palpation | Not reported |
|  | Strength (MMT, DNF endurance) | Not reported |
|  | Joint position error test | Adults with cervical spine injury:  **Sensitivity:** 82%  **Specificity:** 90%  [14] |

BESS = balance error scoring system; DNF = deep neck flexor; mBESS = modified balance error scoring system; MMT=manual muscle testing; MRC = medical research council; TBI = traumatic brain injury; VOMS = vestibular ocular motor screening; VOR = vestibulo-ocular reflex

**References for Table S1**

1. Winser SJ, Smith C, Hale LA, Claydon LS, Whitney SL. Balance outcome measures in cerebellar ataxia: a Delphi survey. *Disability and Rehabilitation*. 2015;37(2):165-170. doi:10.3109/09638288.2014.913709

2. Anderson NE. Detection of focal cerebral hemisphere lesions using the neurological examination. Journal of Neurology, Neurosurgery & Psychiatry; 2005, 76(4):545-549. doi:10.1136/jnnp.2004.043679

3.Büttner F, Howell DR, Doherty C, Blake C, Ryan J, Delahunt E. Clinical detection and recovery of vestibular and oculomotor impairments among amateur athletes following sport-related concussion: A prospective, matched-cohort study. Journal of Head Trauma Rehabilitation; 2021, 36(2):87-95. doi:10.1097/HTR.0000000000000608

4.Omaña H, Bezaire K, Brady K, et al. Functional reach test, single-leg stance test, and tinetti performance-oriented mobility assessment for the prediction of falls in older adults: A systematic review. Physical Therapy; 2021, 101(10):pzab173. doi:10.1093/ptj/pzab173

5.Buckley TA, Munkasy BA, Clouse BP. Sensitivity and specificity of the modified balance error scoring system in concussed collegiate student athletes. Clinical Journal of Sport Medicine; 2018, 28(2):174-176. doi:10.1097/JSM.0000000000000426

6.Haran FJ, Slaboda JC, King LA, Wright WG, Houlihan D, Norris JN. Sensitivity of the balance error scoring system and the sensory organization test in the combat environment. Journal of Neurotrauma; 2016, 33(7):705-711. doi:10.1089/neu.2015.4060

7.Bhattacharyya N, Gubbels SP, Schwartz SR, et al. Clinical practice guideline: Benign paroxysmal positional vertigo (update). Otolaryngol Head Neck Surg; 2017, 156(3_suppl):S1-S47. doi:10.1177/0194599816689667

8.Jorns-Haderli M, Straumann D, Palla A. Accuracy of the bedside head impulse test in detecting vestibular hypofunction. Journal of Neurology, Neurosurgery & Psychiatry; 2007, 78(10):1113-1118. doi:10.1136/jnnp.2006.109512

9.Corwin DJ, Mcdonald CC, Arbogast KB, et al. Clinical and device-based metrics of gait and balance in diagnosing youth concussion. Medicine & Science in Sports & Exercise; 2020, 52(3):542-548. doi:10.1249/MSS.0000000000002163

10.Murray N, Salvatore A, Powell D, Reed-Jones R. Reliability and validity evidence of multiple balance assessments in athletes with a concussion. Journal of Athletic Training; 2014, 49(4):540-549. doi:10.4085/1062-6050-49.3.32

11.Hunfalvay M, Roberts CM, Murray N, Tyagi A, Kelly H, Bolte T. Horizontal and vertical self-paced saccades as a diagnostic marker of traumatic brain injury. Concussion; 2019, 4(1):CNC60. doi:10.2217/cnc-2019-0001

12.Hunfalvay M, Roberts CM, Murray NP, et al. Vertical smooth pursuit as a diagnostic marker of traumatic brain injury. Concussion; 2020, 5(1):CNC69. doi:10.2217/cnc-2019-0013

13.Dall’Alba PT, Sterling MM, Treleaven JM, Edwards SL, Jull GA. Cervical range of motion discriminates between asymptomatic persons and those with whiplash. Spine; 2001, 26(19):2090-2094. doi:10.1097/00007632-200110010-00009

14.Cheever K, Kawata K, Tierney R, Galgon A. Cervical injury assessments for concussion evaluation: A review. Journal of Athletic Training; 2016, 51(12):1037-1044. doi:10.4085/1062-6050-51.12.15
