# Supplementary material for: Clinician-Prioritized Measures to Use in a Remote Concussion Assessment: Delphi Study
Source: JMIR Form Res. 2024 Sep 2;8:e47246. doi: 10.2196/47246 (PMC11406108; doi:10.2196/47246)
Supplement: Multimedia Appendix 2 [file formative_v8i1e47246_app2.docx]

**Multimedia Appendix 2.** Delphi survey round one: measures that did not meet the 15% cut-off criteria.

**Table S2**. Clinician-identified measures that did not meet the 15% cut-off criteria in Delphi survey round one (N=58).

| **Domain** | **Measures** | **Frequency** | **Agreement**  **(N/58*100)** |
| --- | --- | --- | --- |
|  |  |  |  |
| **Neurological Examination** | Functional testing/capacity | 5 | 9% |
|  | Babinski, Hoffman's | 4 | 7% |
|  | Infrared Goggle Testing | 3 | 5% |
|  | Observation, Auditory screen, Dysmetria, Dysdiaokokinesia, Clonus, Upper limb tension tests, Mental capacity/status | 2 | 3% |
|  | Adiadokokinesis, Calibrated stick drop reaction time, Fukuda step test, Upper motor neuron testing, Hyperventilation, Verbal screen, Plantar reflex, Optic nerve/disc assessment, Cognition, Temporomandibular joint range of motion, Mini-mental state examination, Higher cortical functions, Tremor, Bradykinesia | 1 | 2% |
| **Vestibular** | Dynamic gait index | 7 | 12% |
|  | Dizziness handicap inventory | 6 | 10% |
|  | Benign paroxysmal positional vertigo assessment, Head roll test | 5 | 9% |
|  | Functional gait assessment | 4 | 7% |
|  | Motion sensitivity testing, Motion sensitivity quotient, Modified clinical test of sensory interaction in balance (mCTSIB) | 3 | 5% |
|  | Head shake, Mini balance evaluation systems test (MiniBest), Berg, Dynamic balance, Walking on heels, Walking on toes | 2 | 3% |
|  | Otoscopy, Community balance and mobility scale (CB&M), Head impulse nystagmus test of skew examination (HINTS), Gait backwards, Gait sideways, Tandem backwards, Grapevine, Computerized dynamic foot plate, Effects of noise, Pressure (Valsalava, tragal pressure) on symptoms, 10m walk test, modified COBALT, Positional testing (with goggles), Narrow base of support with eyes closed, Epleys, Peripheral vestibular assessment, Visual vertigo analogue score, Provoked vertigo (supine to sit/sit to supine, bend to floor) | 1 | 2% |
| **Oculomotor** | Cover eye test/cover/uncover test, Visual fields | 8 | 14% |
|  | Pupil reactivity, Accommodation, Tracking, Nystagmus check | 7 | 12% |
|  | Gaze stabilization | 6 | 10% |
|  | Visual motion sensitivity, Extraocular muscles (EOM) | 5 | 9% |
|  | King-Devick, Visual midline shift | 4 | 7% |
|  | Visual acuity, Fixation | 2 | 3% |
|  | Pupils equal round reactive to light (PERL), Single and 2-point gaze, Optic discs, Diplopia, Focus, Skew deviation, Eyeball movement, Light and accommodations reflex, Subjective visual vertical test, University of Pennsylvania hand out | 1 | 2% |
| **Cervical** | Vertebral artery assessment | 8 | 14% |
|  | Spurlings | 7 | 12% |
|  | Proprioception, Flexion rotation test | 6 | 10% |
|  | Alar & transverse ligament tests, Passive intervertebral movements/Passive accessory intervertebral movement ( PIVM/PAIVMs), Posture | 4 | 7% |
|  | Tenderness, Deep neck extensor, Joint mobility | 3 | 5% |
|  | Dynamic stability, Smooth pursuit neck torsion test, Cranio-cervical stability | 2 | 3% |
|  | Neck muscle length/tension, Localized pain, Cervical axial compression muscle flexibility, Trigger point assessment, Times testing for neck endurance, Muscle tone and control, Head neck dissociation, Upper limb scan, Dynamic mobility, Head lift endurance, Neck extension rotation test, Joint glides, L’hermitte, RIMS, Upper extremity tests, Muscle bulk activity, Upper extremity strength, Provocative maneuvers for cervicogenic headache, Craniovertebral ligament stability, X-ray cervical spine | 1 | 2% |
| **Effort** | None | 31 | 53% |
|  | Buffalo Concussion Treadmill Test | 10 | 17% |
|  | Buffalo Concussion Bike Test, Functional lifting | 4 | 7% |
|  | Observation, Immediate post-concussion assessment and cognitive test (ImPACT), Consistency of presentation/Abnormal responses, Treadmill test | 3 | 5% |
|  | Waddel sign | 2 | 3% |
|  | Rey’s 15 item test, Attentional test, RPE, Symptom scales | 1 | 2% |
