# Supplementary material for: Clinician-Prioritized Measures to Use in a Remote Concussion Assessment: Delphi Study
Source: JMIR Form Res. 2024 Sep 2;8:e47246. doi: 10.2196/47246 (PMC11406108; doi:10.2196/47246)
Supplement: Multimedia Appendix 3 [file formative_v8i1e47246_app3.docx]

**Multimedia Appendix 3.** Round 2 mean rankings.

**Table S3**. Delphi survey round two mean rankings for each measure.

|  | | **Mean Rankings** | | **Response Count and Weight** | | | | | | | | | | | | | | | | | | | | | | | | | | |  |
| --- | --- | --- | --- | --- | --- | --- | --- | --- | --- | --- | --- | --- | --- | --- | --- | --- | --- | --- | --- | --- | --- | --- | --- | --- | --- | --- | --- | --- | --- | --- | --- |
|  |  |  |  |  |  |  |  |  |  |  |  |  |  |  |  |  |  |  |  |  |  |  |  |  |  |  |  |  |  |  |  |
|  |  |  |  | **Rank 1** | | | **Rank 2** | | | **Rank 3** | | | **Rank 4** | | | **Rank 5** | | | **Rank 6** | | | **Rank 7** | | | **Rank 8** | | | **Rank 9** | | |  |
| **Domain** | **Measures** | **Mean Ranking** | **Sum of all (weight*response count)** | **Frequency (#)** | **Weight** | **Weight*Response Count** | **Frequency (#)** | **Weight** | **Weight*Response Count** | **Frequency (#)** | **Weight** | **Weight*Response Count** | **Frequency (#)** | **Weight** | **Weight*Response Count** | **Frequency (#)** | **Weight** | **Weight*Response Count** | **Frequency (#)** | **Weight** | **Weight*Response Count** | **Frequency (#)** | **Weight** | **Weight*Response Count** | **Frequency (#)** | **Weight** | **Weight*Response Count** | **Frequency (#)** | **Weight** | **Weight*Response Count** |  |
| **Neurological Examination** | Cerebellar testing (Coordination: finger-to-nose, heel-to-shin; Rapid alternating movements) | 4.666666667 | 154 | 10 | 6 | 60 | 16 | 5 | 80 | 0 | 4 | 0 | 2 | 3 | 6 | 4 | 2 | 8 | 0 | 1 | 0 | N/A | N/A | N/A | N/A | N/A | N/A | N/A | N/A | N/A |  |
|  | Cranial nerve | 4.515151515 | 149 | 17 | 6 | 102 | 4 | 5 | 20 | 3 | 4 | 12 | 1 | 3 | 3 | 4 | 2 | 8 | 4 | 1 | 4 | N/A | N/A | N/A | N/A | N/A | N/A | N/A | N/A | N/A |  |
|  | Sensation | 2.424242424 | 80 | 0 | 6 | 0 | 1 | 5 | 5 | 6 | 4 | 24 | 7 | 3 | 21 | 11 | 2 | 22 | 8 | 1 | 8 | N/A | N/A | N/A | N/A | N/A | N/A | N/A | N/A | N/A |  |
|  | Reflexes | 2.848484848 | 94 | 2 | 6 | 12 | 1 | 5 | 5 | 6 | 4 | 24 | 10 | 3 | 30 | 9 | 2 | 18 | 5 | 1 | 5 | N/A | N/A | N/A | N/A | N/A | N/A | N/A | N/A | N/A |  |
|  | Motor (tone, pronator drift, strength/power using MRC grading/MMT) | 4.090909091 | 135 | 4 | 6 | 24 | 8 | 5 | 40 | 14 | 4 | 56 | 3 | 3 | 9 | 1 | 2 | 2 | 4 | 1 | 4 | N/A | N/A | N/A | N/A | N/A | N/A | N/A | N/A | N/A |  |
|  | Myotomes | 2.454545455 | 81 | 0 | 6 | 0 | 3 | 5 | 15 | 4 | 4 | 16 | 10 | 3 | 30 | 4 | 2 | 8 | 12 | 1 | 12 | N/A | N/A | N/A | N/A | N/A | N/A | N/A | N/A | N/A |  |
| **Vestibular** | VOMS | 7.515151515 | 248 | 17 | 9 | 153 | 3 | 8 | 24 | 3 | 7 | 21 | 5 | 6 | 30 | 2 | 5 | 10 | 1 | 4 | 4 | 2 | 3 | 6 | 0 | 2 | 0 | 0 | 1 | 0 |  |
|  | Balance (feet together, single leg stance, tandem stance) | 6.454545455 | 213 | 5 | 9 | 45 | 11 | 8 | 88 | 4 | 7 | 28 | 6 | 6 | 36 | 1 | 5 | 5 | 0 | 4 | 0 | 1 | 3 | 3 | 3 | 2 | 6 | 2 | 1 | 2 |  |
|  | VOR test | 6.181818182 | 204 | 1 | 9 | 9 | 9 | 8 | 72 | 6 | 7 | 42 | 5 | 6 | 30 | 7 | 5 | 35 | 3 | 4 | 12 | 1 | 3 | 3 | 0 | 2 | 0 | 1 | 1 | 1 |  |
|  | BESS/mBESS | 5.606060606 | 185 | 2 | 9 | 18 | 4 | 8 | 32 | 6 | 7 | 42 | 7 | 6 | 42 | 3 | 5 | 15 | 5 | 4 | 20 | 4 | 3 | 12 | 2 | 2 | 4 | 0 | 1 | 0 |  |
|  | Dix-Hallpike | 4.575757576 | 151 | 3 | 9 | 27 | 1 | 8 | 8 | 2 | 7 | 14 | 5 | 6 | 30 | 8 | 5 | 40 | 2 | 4 | 8 | 5 | 3 | 15 | 2 | 2 | 4 | 5 | 1 | 5 |  |
|  | Head thrust/Head impulse test | 3.909090909 | 129 | 2 | 9 | 18 | 1 | 8 | 8 | 5 | 7 | 35 | 1 | 6 | 6 | 3 | 5 | 15 | 4 | 4 | 16 | 5 | 3 | 15 | 4 | 2 | 8 | 8 | 1 | 8 |  |
|  | Gait/Tandem gait | 4.363636364 | 144 | 3 | 9 | 27 | 1 | 8 | 8 | 5 | 7 | 35 | 3 | 6 | 18 | 1 | 5 | 5 | 7 | 4 | 28 | 3 | 3 | 9 | 4 | 2 | 8 | 6 | 1 | 6 |  |
|  | Romberg | 2.878787879 | 95 | 0 | 9 | 0 | 0 | 8 | 0 | 1 | 7 | 7 | 1 | 6 | 6 | 6 | 5 | 30 | 1 | 4 | 4 | 9 | 3 | 27 | 6 | 2 | 12 | 9 | 1 | 9 |  |
|  | Dynamic Visual Acuity | 3.515151515 | 116 | 0 | 9 | 0 | 3 | 8 | 24 | 1 | 7 | 7 | 0 | 6 | 0 | 2 | 5 | 10 | 10 | 4 | 40 | 3 | 3 | 9 | 12 | 2 | 24 | 2 | 1 | 2 |  |
| **Oculomotor** | Saccades | 1.909090909 | 63 | 7 | 3 | 21 | 16 | 2 | 32 | 10 | 1 | 10 | N/A | N/A | N/A | N/A | N/A | N/A | N/A | N/A | N/A | N/A | N/A | N/A | N/A | N/A | N/A | N/A | N/A | N/A |  |
|  | Convergence | 1.848484848 | 61 | 11 | 3 | 33 | 6 | 2 | 12 | 16 | 1 | 16 | N/A | N/A | N/A | N/A | N/A | N/A | N/A | N/A | N/A | N/A | N/A | N/A | N/A | N/A | N/A | N/A | N/A | N/A |  |
|  | Smooth pursuits | 2.242424242 | 74 | 15 | 3 | 45 | 11 | 2 | 22 | 7 | 1 | 7 | N/A | N/A | N/A | N/A | N/A | N/A | N/A | N/A | N/A | N/A | N/A | N/A | N/A | N/A | N/A | N/A | N/A | N/A |  |
| **Cervical** | Range of motion | 3.666666667 | 121 | 25 | 4 | 100 | 6 | 3 | 18 | 1 | 2 | 2 | 1 | 1 | 1 | N/A | N/A | N/A | N/A | N/A | N/A | N/A | N/A | N/A | N/A | N/A | N/A | N/A | N/A | N/A |  |
|  | Palpation | 2.939393939 | 97 | 7 | 4 | 28 | 20 | 3 | 60 | 3 | 2 | 6 | 3 | 1 | 3 | N/A | N/A | N/A | N/A | N/A | N/A | N/A | N/A | N/A | N/A | N/A | N/A | N/A | N/A | N/A |  |
|  | Strength (MMT, DNF endurance) | 1.878787879 | 62 | 0 | 4 | 0 | 2 | 3 | 6 | 25 | 2 | 50 | 6 | 1 | 6 | N/A | N/A | N/A | N/A | N/A | N/A | N/A | N/A | N/A | N/A | N/A | N/A | N/A | N/A | N/A |  |
|  | Joint position error test | 1.515151515 | 50 | 1 | 4 | 4 | 5 | 3 | 15 | 4 | 2 | 8 | 23 | 1 | 23 | N/A | N/A | N/A | N/A | N/A | N/A | N/A | N/A | N/A | N/A | N/A | N/A | N/A | N/A | N/A |  |
