# Supplementary material for: Clinician-Prioritized Measures to Use in a Remote Concussion Assessment: Delphi Study
Source: JMIR Form Res. 2024 Sep 2;8:e47246. doi: 10.2196/47246 (PMC11406108; doi:10.2196/47246)
Supplement: Multimedia Appendix 4 [file formative_v8i1e47246_app4.docx]

**Multimedia Appendix 4.** Round 2 consensus values.

While the aim of the study was to identify the most relevant measures using rank-orders of measures, consensus values for rankings of each measure were also calculated. Frequency counts and consensus values (calculated by dividing frequency counts by number of responses for each ranking) for measures that reached or exceeded 50% consensus are shown in table S4. The consensus target, greater than 50%, was reached on the first-ranked measure for three of the five domains. For the neurological examination and vestibular domains, consensus at 51.52% was reached for the first ranked outcome measures of cranial nerve evaluation and VOMS, respectively. The cervical spine assessment was the only domain where consensus was reached for all measures (range of motion, followed by palpation, followed by strength, followed by the joint position error test) with range of motion ranked as most relevant at 75.76%. There was no consensus on the oculomotor domain measures.

**Table S4**. Frequency counts and consensus values for measures reaching at least 50% consensus within each rank.

|  | | **Frequency (#) and Consensus (%)** | | | | | | | |
| --- | --- | --- | --- | --- | --- | --- | --- | --- | --- |
|  |  | **Rank 1** | | **Rank 2** | | **Rank 3** | | **Rank 4** | |
| **Domain** | **Measures** | **#** | **%** | **#** | **%** | **#** | **%** | **#** | **%** |
| **Neurological Examination** | Coordination: finger-to-nose, heel-to-shin; Rapid alternating movements | - | - | - | - | - | - | - | - |
|  | Cranial nerve | 17 | 51.52 | - | - | - | - | - | - |
|  | Motor (tone, pronator drift, strength/power using MRC grading/MMT) | - | - | - | - | - | - | - | - |
| **Vestibular** | VOMS | 17 | 51.52 | - | - | - | - | - | - |
| **Cervical** | Range of motion | 25 | 75.76 | - | - | - | - | - | - |
|  | Palpation | - | - | 20 | 60.61 | - | - | - | - |
|  | Strength (MMT, DNF endurance) | - | - | - | - | 25 | 75.76 | - | - |
|  | Joint position error test | - | - | - | - | - | - | 23 | 69.70 |

MMT=manual muscle testing; MRC = medical research council

Frequency counts and consensus values for all measures that met the previously set criteria (15% of clinicians identifying a measure) are presented in Table S5.

**Table S5**. Delphi survey round 2 frequency counts and consensus values for all measures that met the previously set criteria.

|  | | **Frequency (#) and Consensus (%)** | | | | | | | | | | | | | | | | | |
| --- | --- | --- | --- | --- | --- | --- | --- | --- | --- | --- | --- | --- | --- | --- | --- | --- | --- | --- | --- |
|  |  |  |  |  |  |  |  |  |  |  |  |  |  |  |  |  |  |  |  |
|  | | **Rank 1** | | **Rank 2** | | **Rank 3** | | **Rank 4** | | **Rank 5** | | **Rank 6** | | **Rank 7** | | **Rank 8** | | **Rank 9** | |
| **Domain** | **Measures** | **#** | **%** | **#** | **%** | **#** | **%** | **#** | **%** | **#** | **%** | **#** | **%** | **#** | **%** | **#** | **%** | **#** | **%** |
| **Neurological Examination** | Cerebellar testing (Coordination: finger-to-nose, heel-to-shin; Rapid alternating movements) | 10 | 30.30 | 16 | 48.48 | 0 | 0.00 | 2 | 6.06 | 4 | 12.12 | 0 | 0.00 | N/A | N/A | N/A | N/A | N/A | N/A |
|  | Cranial nerve | 17 | 51.52 | 4 | 12.12 | 3 | 9.09 | 1 | 3.03 | 4 | 12.12 | 4 | 12.12 | N/A | N/A | N/A | N/A | N/A | N/A |
|  | Sensation | 0 | 0.00 | 1 | 3.03 | 6 | 18.18 | 7 | 21.21 | 11 | 33.33 | 8 | 24.24 | N/A | N/A | N/A | N/A | N/A | N/A |
|  | Reflexes | 2 | 6.06 | 1 | 3.03 | 6 | 18.18 | 10 | 30.30 | 9 | 27.27 | 5 | 15.15 | N/A | N/A | N/A | N/A | N/A | N/A |
|  | Motor (tone, pronator drift, strength/power using MRC grading/MMT) | 4 | 12.12 | 8 | 24.24 | 14 | 42.42 | 3 | 9.09 | 1 | 3.03 | 4 | 12.12 | N/A | N/A | N/A | N/A | N/A | N/A |
|  | Myotomes | 0 | 0.00 | 3 | 9.09 | 4 | 12.12 | 10 | 30.30 | 4 | 12.12 | 12 | 36.36 | N/A | N/A | N/A | N/A | N/A | N/A |
| **Vestibular** | VOMS | 17 | 51.52 | 3 | 9.09 | 3 | 9.09 | 5 | 15.15 | 2 | 6.06 | 1 | 3.03 | 2 | 6.06 | 0 | 0.00 | 0 | 0.00 |
|  | Balance (feet together, single leg stance, tandem stance) | 5 | 15.15 | 11 | 33.33 | 4 | 12.12 | 6 | 18.18 | 1 | 3.03 | 0 | 0.00 | 1 | 3.03 | 3 | 9.09 | 2 | 6.06 |
|  | VOR test | 1 | 3.03 | 9 | 27.27 | 6 | 18.18 | 5 | 15.15 | 7 | 21.21 | 3 | 9.09 | 1 | 3.03 | 0 | 0.00 | 1 | 3.03 |
|  | BESS/mBESS | 2 | 6.06 | 4 | 12.12 | 6 | 18.18 | 7 | 21.21 | 3 | 9.09 | 5 | 15.15 | 4 | 12.12 | 2 | 6.06 | 0 | 0.00 |
|  | Dix-Hallpike | 3 | 9.09 | 1 | 3.03 | 2 | 6.06 | 5 | 15.15 | 8 | 24.24 | 2 | 6.06 | 5 | 15.15 | 2 | 6.06 | 5 | 15.15 |
|  | Head thrust/Head impulse test | 2 | 6.06 | 1 | 3.03 | 5 | 15.15 | 1 | 3.03 | 3 | 9.09 | 4 | 12.12 | 5 | 15.15 | 4 | 12.12 | 8 | 24.24 |
|  | Gait/Tandem gait | 3 | 9.09 | 1 | 3.03 | 5 | 15.15 | 3 | 9.09 | 1 | 3.03 | 7 | 21.21 | 3 | 9.09 | 4 | 12.12 | 6 | 18.18 |
|  | Romberg | 0 | 0.00 | 0 | 0.00 | 1 | 3.03 | 1 | 3.03 | 6 | 18.18 | 1 | 3.03 | 9 | 27.27 | 6 | 18.18 | 9 | 27.27 |
|  | Dynamic Visual Acuity | 0 | 0.00 | 3 | 9.09 | 1 | 3.03 | 0 | 0.00 | 2 | 6.06 | 10 | 30.30 | 3 | 9.09 | 12 | 36.36 | 2 | 6.06 |
| **Oculomotor** | Saccades | 7 | 21.21 | 16 | 48.48 | 10 | 30.30 | N/A | N/A | N/A | N/A | N/A | N/A | N/A | N/A | N/A | N/A | N/A | N/A |
|  | Convergence | 11 | 33.33 | 6 | 18.18 | 16 | 48.48 | N/A | N/A | N/A | N/A | N/A | N/A | N/A | N/A | N/A | N/A | N/A | N/A |
|  | Smooth pursuits | 15 | 45.45 | 11 | 33.33 | 7 | 21.21 | N/A | N/A | N/A | N/A | N/A | N/A | N/A | N/A | N/A | N/A | N/A | N/A |
| **Cervical** | Range of motion | 25 | 75.76 | 6 | 18.18 | 1 | 3.03 | 1 | 3.03 | N/A | N/A | N/A | N/A | N/A | N/A | N/A | N/A | N/A | N/A |
|  | Palpation | 7 | 21.21 | 20 | 60.61 | 3 | 9.09 | 3 | 9.09 | N/A | N/A | N/A | N/A | N/A | N/A | N/A | N/A | N/A | N/A |
|  | Strength (MMT, DNF endurance) | 0 | 0.00 | 2 | 6.06 | 25 | 75.76 | 6 | 18.18 | N/A | N/A | N/A | N/A | N/A | N/A | N/A | N/A | N/A | N/A |
|  | Joint position error test | 1 | 3.03 | 5 | 15.15 | 4 | 12.12 | 23 | 69.70 | N/A | N/A | N/A | N/A | N/A | N/A | N/A | N/A | N/A | N/A |
